# Cardiac and Vascular Surgery–Associated Acute Kidney Injury: The 20th International Consensus Conference of the ADQI (Acute Disease Quality Initiative) Group

**DOI:** 10.1161/JAHA.118.008834

**Published:** 2018-06-01

**Authors:** Mitra K. Nadim, Lui G. Forni, Azra Bihorac, Charles Hobson, Jay L. Koyner, Andrew Shaw, George J. Arnaoutakis, Xiaoqiang Ding, Daniel T. Engelman, Hrvoje Gasparovic, Vladimir Gasparovic, Charles A. Herzog, Kianoush Kashani, Nevin Katz, Kathleen D. Liu, Ravindra L. Mehta, Marlies Ostermann, Neesh Pannu, Peter Pickkers, Susanna Price, Zaccaria Ricci, Jeffrey B. Rich, Lokeswara R. Sajja, Fred A. Weaver, Alexander Zarbock, Claudio Ronco, John A. Kellum

**Affiliations:** ^1^ Division of Nephrology & Hypertension Department of Medicine Keck School of Medicine University of Southern California Los Angeles CA; ^2^ Division of Vascular Surgery Department of Surgery Keck School of Medicine University of Southern California Los Angeles CA; ^3^ Department of Clinical & Experimental Medicine University of Surrey Guildford United Kingdom; ^4^ Royal Surrey County Hospital NHS Foundation Trust Guildford United Kingdom; ^5^ Division of Nephrology, Hypertension & Renal Transplantation Department of Medicine University of Florida Gainesville FL; ^6^ Division of Surgical Critical Care Department of Surgery Malcom Randall VA Medical Center Gainesville FL; ^7^ Section of Nephrology Department of Medicine University of Chicago IL; ^8^ Department of Anesthesiology Vanderbilt University Medical Center Nashville TN; ^9^ Division of Thoracic & Cardiovascular Surgery Department of Surgery University of Florida College of Medicine Gainesville FL; ^10^ Department of Nephrology Shanghai Institute for Kidney Disease and Dialysis Shanghai Medical Center for Kidney Disease Zhongshan Hospital Fudan University Shanghai China; ^11^ Division of Cardiac Surgery Department of Surgery Baystate Medical Center University of Massachusetts Medical School Springfield MA; ^12^ Department of Cardiac Surgery University Hospital Rebro Zagreb Croatia; ^13^ Department of Medicine University of Zagreb Croatia; ^14^ Division of Cardiology Department of Medicine Hennepin County Medical Center University of Minnesota Minneapolis MN; ^15^ Division of Nephrology & Hypertension Division of Pulmonary and Critical Care Medicine Department of Medicine Mayo Clinic Rochester MN; ^16^ Division of Cardiac Surgery Department of Surgery Johns Hopkins University Baltimore MD; ^17^ Divisions of Nephrology and Critical Care Departments of Medicine and Anesthesia University of California San Francisco CA; ^18^ Department of Medicine UCSD Medical Center University of California San Diego CA; ^19^ King's College London Guy's & St Thomas’ Hospital London United Kingdom; ^20^ Division of Critical Care Medicine Faculty of Medicine and Dentistry University of Alberta Edmonton Canada; ^21^ Department Intensive Care Medicine Radboud University Medical Center Nijmegen The Netherlands; ^22^ Adult Intensive Care Unit Imperial College Royal Brompton Hospital London United Kingdom; ^23^ Department of Pediatric Cardiac Surgery Bambino Gesù Children's Hospital Roma Italy; ^24^ Heart and Vascular Institute Cleveland Clinic Cleveland OH; ^25^ Division of Cardiothoracic Surgery STAR Hospitals Hyderabad India; ^26^ Department of Anesthesiology, Intensive Care and Pain Medicine University Hospital Münster Münster Germany; ^27^ Department of Nephrology, Dialysis and Transplantation San Bortolo Hospital International Renal Research Institute of Vicenza Italy; ^28^ Department of Critical Care Medicine School of Medicine University of Pittsburgh PA

**Keywords:** biomarker, dialysis, diuretics, ischemia–reperfusion injury, renal insufficiency, Statements and Guidelines, Vascular Disease, Cardiovascular Surgery, Primary Prevention, Treatment

## Introduction

Acute kidney injury (AKI) occurs in 7% to 18% of hospitalized patients and complicates the course of 50% to 60% of those admitted to the intensive care unit, carrying both significant mortality and morbidity.^1^ Even though many cases of AKI are reversible within days to weeks of occurrence, data from multiple large observational and epidemiological studies over the past decade suggest a strong association between AKI and subsequent chronic kidney disease (CKD) and end‐stage renal disease (ESRD).^2^, ^3^ Patients with AKI who receive renal replacement therapy (RRT) are >3 times more likely to develop ESRD than those who do not. This rise in the number of patients who receive treatment for ESRD is a global phenomenon associated with considerable patient costs, effects on quality of life, and economic impact on society as a whole. In developing countries, most people with kidney failure have insufficient access to dialysis and/or kidney transplantation. Consequently, the development of effective approaches to the prevention, early recognition, and management of AKI is necessary to reduce the burden of CKD and ESRD.^4^

Millions of patients undergo cardiac and vascular surgery (CVS) every year in developed countries alone. AKI is a common perioperative complication for patients undergoing both cardiac surgery^5^, ^6^, ^7^, ^8^, ^9^ and vascular surgery,^9^, ^10^, ^11^ occurring in 20% to 70% of cases depending on the type of surgery and the definition of AKI used. In addition, more and more of these patients who receive complex CVS are elderly with multiple comorbidities, which predispose to the development of AKI and potentially hasten progression to ESRD. Mortality rates among cardiovascular patients undergoing RRT are between 40% and 70%, and mortality is associated with both the severity of the initial insult and the number of episodes of AKI occurring during the hospital admission.^12^, ^13^

In recent years, there have been considerable advances in our understanding of CVS‐associated AKI (CVS‐AKI). Nevertheless, despite the high prevalence, there is little consensus about how best to prevent or treat CVS‐AKI. The aim of this consensus process was to review the current literature on CVS‐AKI; to create the basis for its definition; to develop an initial understanding of its pathophysiology; to explore the potential use of biomarkers for its diagnosis; to critique current literature in the fields of prevention and treatment, so as to make recommendations for clinical practice; and to propose a framework for future research.

## Methods

ADQI (Acute Disease Quality Initiative) is an ongoing process that produces evidence‐based recommendations on the diagnosis, prevention, and management of AKI and on various issues concerning acute dialysis and fluid management (http://www.adqi.org). The conference chairs of the 20th ADQI consensus committee (M.K.N., J.A.K., V.G., C.R., and L.G.F.) convened a panel of experts representing the relevant disciplines—cardiac surgery, vascular surgery, cardiology, nephrology, anesthesiology, and critical care—from North America, Europe, and Asia to discuss the issues related to CVS‐AKI (Data S1). The conference took place June 16 to 19, 2017, and the format of the meeting was a 2.5‐day modified Delphi method to achieve consensus, as described previously (Data S1).^14^

## Results and Discussion

### Pathophysiology

**Figure 1:**
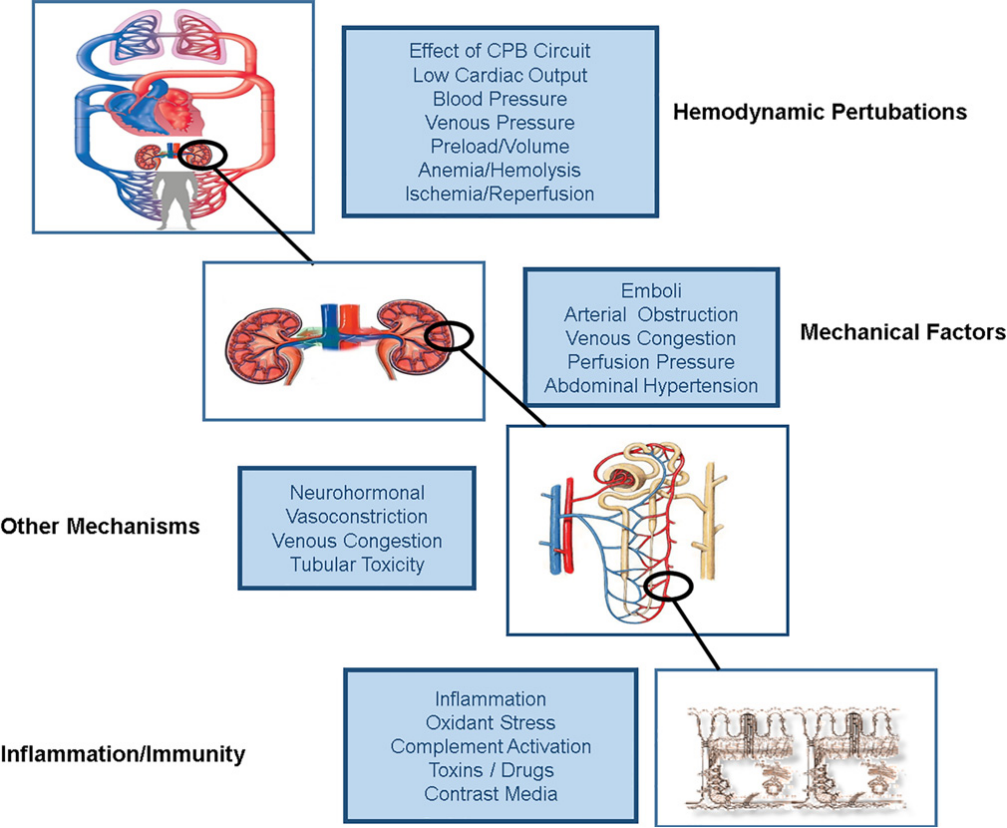
Major pathophysiological mechanisms for the development of cardiac and vascular surgery–associated acute kidney injury (CVS‐AKI). Many common factors contribute to the development of CVS‐AKI. Hemodynamic perturbations such as exposure to cardiopulmonary bypass (CPB), cross‐clamping of the aorta, high doses of exogenous vasopressors, and blood‐product transfusion all increase the risk of AKI. Similarly, the mechanical factors outlined may be associated with renal perfusion injury following episodes of ischemia, resulting in increased oxidative stress and associated inflammation as well as embolic disease including cholesterol emboli, all of which increase the pathological burden on the kidney. Other mechanisms such as neurohormonal activation are relevant, as is the generation of free hemoglobin and the liberation of free iron perioperatively, all potentiating AKI. Associated tissue damage is reflected in a systemic inflammatory response, and all these factors contribute to a significant inflammatory response. Immune activation, the generation of reactive oxygen species, and upregulation of proinflammatory transcription factors all play roles.

The pathophysiology of CVS‐AKI is complex and poorly understood. Although patients undergoing cardiac and major vascular surgery may experience similar insults to the kidneys, many distinctions exist between these populations. Notable differences are the relative influence of cardiac dysfunction (greater in cardiac surgery) versus warm ischemia–reperfusion injury to the kidneys and increased abdominal pressures (both greater with vascular surgery). Finally, the effect of the cardiopulmonary bypass (CPB) circuit itself in the case of cardiac surgery is notable. Although animal models^15^, ^16^ of CPB and cardiac surgery–associated AKI (CS‐AKI) exist, they have not been widely applied to the study of AKI. Furthermore, although clinical studies have been conducted for >40 years, numerous knowledge gaps remain. Observational studies, animal and cell culture work, and mathematical simulations^17^ are currently available to predict the events likely to occur during cardiac surgery. Hemodynamic disturbances at each level of arterial blood supply dominate the discussion, and inflammatory, immunological, neurohumoral, and mechanical factors are also of significance (Figure 1).

#### Hemodynamic perturbations

Perturbations in the renal blood flow may lead to an imbalance of oxygen supply and demand.^18^, ^19^ The inner stripe of the outer medullary portion of the kidney may be susceptible to ischemic damage caused by low resting po_2_ (10–20 mm Hg).^18^ During CPB, cardiac output is preserved, but the target blood pressure under such nonpulsatile conditions is unknown, and inadequate renal perfusion may contribute to AKI. However, using a mathematical model,^17^ the rewarming phase of CPB appeared to represent the period when the renal medulla may be at most risk because of the combination of high oxygen demand and low oxygen supply occurring at this time. Low cardiac output states during and after cardiac surgery are likely to contribute to the ischemic process, although whether low flow, low blood pressure, or oxygen delivery is the main culprit remains elusive.^20^ Studies of noncardiac surgery patients suggest that maintenance of sufficient mean arterial pressure is the most important hemodynamic parameter to preserve in the perioperative period^21^, ^22^; however, these patients are rarely exposed to hypothermia and hemodilution, so it remains unclear whether this finding also applies to cardiac surgery patients.

The period after CPB may be relevant for the development of reperfusion injury,^23^ and the precise underlying mechanisms need to be fully understood so that preventive and salvage treatments may be developed to mitigate this process. Remote ischemic preconditioning (RIPC) appeared to show great promise in a study of high‐risk patients^24^ but has been shown to be ineffective (at least with respect to the effect sizes examined) in lower risk patients.^25^, ^26^, ^27^ Some controversy exists regarding the effects of propofol, which has been hypothesized to attenuate the response to RIPC.^26^

The role of venous congestion in the development of AKI is a potential area of pathophysiological significance.^28^, ^29^ The role of high central venous pressure in congestive heart failure is well appreciated.^30^ The incidence of AKI in this population has led investigators to study it in the context of heart surgery, for which the problem is typically in the right heart, and vascular surgery, for which the problem may be increased abdominal compartment pressures. However, the mechanisms involved are unclear, and although “back pressure” on the glomerular apparatus has been postulated, it is unlikely that this process is the sole cause of this observation. It may be that the renal pelvis is able to compensate for a certain amount of increased venous volume before the pressure–flow relationship inside the poorly compliant renal capsule changes, in keeping with the Monro–Kellie doctrine observed in the brain.^31^ Whether this truly applies to the kidney is currently a matter of speculation but one that merits further study.

#### Inflammation and immunity

The systemic inflammatory response is often observed following major surgery, with considerable variability observed between individuals, although it is recognized that a more severe response is associated with an increased risk of adverse outcomes including AKI.^32^, ^33^, ^34^ Unsurprisingly, CVS is often associated with such a response and may activate the inflammatory cascade through several pathways.^35^, ^36^ CVS exposes the patient to a risk profile somewhat different from most other major surgeries. CPB, cross‐clamping of the aorta, high doses of exogenous vasopressors, and high rates of exogenous blood product transfusion, for example, all enhance the risks of AKI, especially when coupled with the risk profile for AKI for most of these patients. Such exposures are associated with perturbations in renal perfusion that induce reperfusion injury following episodes of ischemia, resulting in increased oxidative stress and associated inflammation.^37^, ^38^ This process is exacerbated by the significant shunting within the kidney that results in the renal medulla and corticomedullary junction being relatively hypoxic relative to other tissues.^18^ In cardiac surgery, the entire cardiac output is exposed to an extracorporeal circuit, and this provides a further inflammatory insult through contact activation from the exposure of blood to the CPB circuit; although in the modern CPB circuit biocompatibility has been optimized, measures of immune activation (cytokine and chemokine levels) increase significantly after CPB.^36^ The generation of reactive oxygen species induces inflammation by upregulation of proinflammatory transcription factors, including NFκ‐B (nuclear factor κ‐B).^39^, ^40^ Cytokines and chemokines recruit neutrophils, macrophages, and lymphocytes into the renal parenchyma. Parenchymal infiltration and activation of these immune cells promote AKI and lead to fibrosis. Avoidance of the CPB machine in an attempt to reduce distant organ function has been successful,^41^ although recently published data suggest that 5‐year survival is lower with off‐pump techniques^42^; this may be a reflection of improved revascularization of the heart with the on‐pump technique. In the presence of concurrent sepsis, such as with bacterial endocarditis, sepsis and surgery appear to be synergistic in terms of affecting an immune response.^43^

#### Iron metabolism and free hemoglobin

CVS leads to free hemoglobin liberation with the release of free iron, and this phenomenon has generated much interest regarding CS‐AKI.^44^, ^45^, ^46^, ^47^ A degree of hemolysis is inevitable whenever red blood cells come into contact with an artificial surface or with air (eg, blood scavenging systems), and this may be coupled with a prolonged period of hypothermia (sometimes as low as 18°C), which creates the perfect environment for hemolysis and liberation of free iron, leading to vasoconstriction through scavenging of nitric oxide by free hemoglobin. Indeed, evidence from a case–control study of patients who developed AKI postoperatively compared with matched controls demonstrated that plasma‐free hemoglobin was less than half that observed in the control group, providing further evidence that hemolysis and free iron may contribute to AKI development.^44^ Moreover, free hemoglobin and, particularly, free ferrous iron increase production of reactive oxygen species via the Fenton and Haber Weis reactions, especially as free hemoglobin and iron are sequestered within the kidney.^48^ Plasma‐free hemoglobin also induces HO‐1 (heme oxygenase 1) expression. HO‐1 degrades heme but increases in experimental models of AKI. Plasma HO‐1 is increased in patients who develop AKI, and CPB duration, hemolysis, and inflammation are associated with increased HO‐1 concentrations following cardiac surgery.^45^

#### Other mechanisms

Oxygen free radical generation and metabolism is an area of active investigation^49^, ^50^ (and genetic predisposition to injury is important^51^, ^52^), but it is not clear whether this results in increased susceptibility to AKI or to innate impairment of the ability to repair and regenerate healthy renal tissue. The precise nature of the genetic (and epigenetic) variables involved also remains unclear. Furthermore, embolic disease is important for CS‐AKI. Cholesterol emboli^53^ are at risk for distal migration when a cross‐clamp is applied or released from the aorta, especially in patients with significant atherosclerosis. Moreover, intra‐aortic balloon counterpulsation devices increase the embolic load, and the fact that these devices are typically deployed in patients with severely compromised hemodynamic conditions makes it difficult to discern whether such devices are of overall benefit (by improving cardiac output) or harm (by increasing generation of emboli) to the kidney. In addition, tissue injury releases mitochondrial damage–associated molecular patterns including mitochondrial DNA, which can act as a direct activator of neutrophils, which in turn elicit a systemic inflammatory response syndrome while suppressing polymorphonuclear function. Such molecular patterns have also been seen during CPB and, as such, may participate in the pathogenesis of CVS‐AKI.^54^

### Diagnosis and Risk Assessment

#### Perioperative stratification for AKI

*Recommendation:*

We recommend routine implementation of validated clinical risk‐prediction models in the preoperative assessment of all patients undergoing CVS, using estimated glomerular filtration rate (eGFR), cystatin C, and/or albuminuria to improve risk stratification of those at intermediate and high risk of AKI postoperatively (not graded).

**Figure 2:**
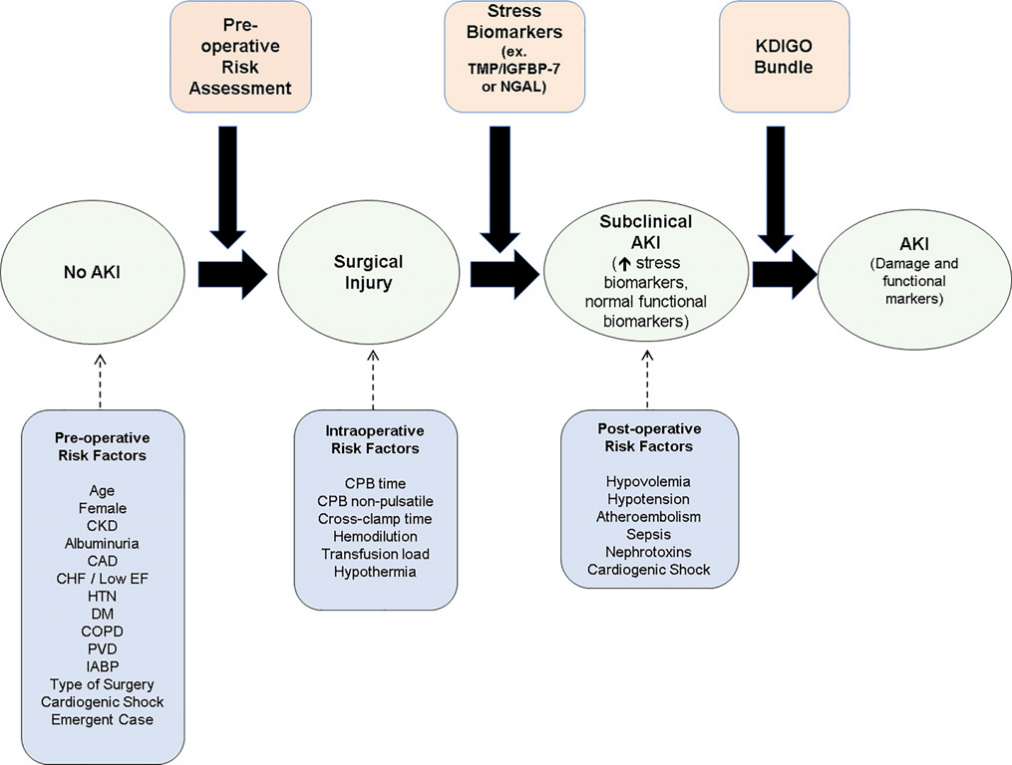
Risk assessment for acute kidney injury (AKI) following cardiac and vascular surgery (CVS). This figure provides a framework for the time course of risk assessment for AKI following CVS. Risk assessment should be a continual process that is repeatedly performed in the pre‐, peri‐, and early postoperative time course, and it should incorporate clinical factors and biomarkers if available. Patients deemed to be at high risk of AKI may benefit from the implementation of kidney‐focused care to improve patient outcomes. CHF indicates congestive heart failure; COPD, chronic obstructive pulmonary disease; CPB, cardiopulmonary bypass; EF, ejection fraction; IABP, intra‐aortic balloon pump; IGFBP7, insulin‐like growth factor binding protein 7; KDIGO, Kidney Disease Initiative Global Outcome; NGAL, neutrophil gelatinase–associated lipocalin; PVD, peripheral vascular disease; TIMP2, tissue inhibitor of metalloproteinases 2.

*Rationale:* Risk assessment is a dynamic process in which patients with fixed preoperative risk derived from underlying comorbidities are evaluated on the basis of additional and potentially modifiable risks from their clinical status before surgery. The use of currently available risk‐prediction instruments must be guided by the goals of risk assessment in each instance. Preoperative risk assessment may be useful for communicating risks associated with surgery to the patient and in implementing preventive strategies in the intra‐ and postoperative periods, for example, goal‐directed hemodynamic management, individualized blood pressure management,^21^ and avoidance of the use of NSAIDs for pain management. Postoperative risk assessment is geared toward early identification of AKI that may allow earlier implementation of preventive strategies. Peri‐ and postoperative risk assessment is geared toward early identification of AKI that may allow proactive treatment. An important conceptual point is that kidney injury in the setting of CVS occurs along a continuum and may relate to patient, preoperative, and intraoperative factors and the trajectory of AKI occurrence from baseline conditions; its development over a patient's clinical course should take this aspect into account (Figure 2).

Although many risk‐prediction scores for AKI after cardiac surgery have been published, only 8 have been externally validated with C statistics ranging from 0.72 to 0.89 (Table S1).^55^, ^56^, ^57^, ^58^, ^59^, ^60^, ^61^ In general, these scoring systems have good discrimination in assessing low‐risk groups but relatively poor discrimination in moderate to high‐risk patients.^62^ There are no externally validated risk‐assessment tools specifically for AKI following vascular surgery; although Kheterpal and colleagues developed and externally validated an AKI risk score for general surgery cases that included but was not limited to vascular surgery.^63^, ^64^ The most robust cardiac surgery prediction tools with the best discrimination have used AKI requiring RRT as an outcome. This is problematic because AKI requiring dialysis, although catastrophic in this context, is relatively uncommon, occurring in 1% to 2% of all patients undergoing surgery in most programs. In addition, the decision to initiate RRT varies among clinicians. Less severe forms of AKI are commonly seen after cardiac surgery with reported rates of Kidney Disease Improving Global Outcomes (KDIGO) stage 1 AKI reported between 20% and 70% depending on the patient risk factors and the inclusion of KDIGO serum creatinine (sCr) and/or urine output (UO) criteria for AKI.^7^, ^8^, ^65^, ^66^, ^67^, ^68^ Three of the prediction rules have assessed risk of less severe forms of AKI defined using sCr criteria only.^56^, ^58^, ^69^ Of these, one used the RIFLE criteria of AKI,^56^ and another used the Acute Kidney Injury Network criteria.^69^

The risk factors commonly identified in externally validated risk‐prediction models are shown in Figure 2. Preexisting CKD, although variably defined, is the strongest risk factor for AKI in this setting. With 2 exceptions,^59^, ^70^ most other prediction tools have used sCr to assess kidney function, which may significantly overestimate kidney function, particularly in malnourished elderly populations. The eGFR, which accounts for age, race, and sex and is subject to similar limitations, is likely a more accurate estimation of kidney function in stable, elective patients. The use of both eGFR and sCr in this context assumes steady‐state kidney function, which is frequently not the case. Newly identified risk factors such as preoperative hemoglobin (anemia/transfusion load) and proteinuria have only been incorporated in recent models, whereas other risk factors have not been rigorously studied for their incremental value when added to existing risk‐prediction models (eg, days from cardiac catheterization to surgery). All risk‐prediction tools have shown only moderate calibration, suggesting significant heterogeneity in the underlying populations.^62^ Because most risk‐prediction tools have been derived from clinical and administrative databases, they fail to capture acuity of illness, which may account for the calibration discrepancies and the difficulty in discrimination among the moderate‐ to high‐risk groups. Some measure or surrogate for hemodynamic stability is present in all published models whether it is characterized by surgical urgency or the presence of cardiogenic shock. It is likely that risk discrimination would improve if additional objectively defined clinical variables were included.

At a minimum, all patients undergoing cardiac and vascular surgical procedures should undergo routine clinical assessment of AKI risk. This involves systematic evaluation of known susceptibilities for development of AKI such as CKD and albuminuria using preoperative sCr and urinalysis in all patients before surgery.^71^, ^72^, ^73^ These results will help frame individualized risk for AKI while perhaps providing insight into patients’ baseline renal function. Whenever possible, efforts should be made to obtain the patient's prior kidney‐function tests to ascertain true baseline function. In summary, the currently available preoperative risk‐assessment tools are beneficial in that they use commonly available data and identify low‐risk patients in the setting of traditional CVS. Nevertheless, they have several limitations:

1. They are predominantly used to predict RRT.
2. Sensitivity and specificity break down at the extremes of the spectrum.
3. They do not account for preoperative eGFR (primarily rely on sCr alone).

Intra‐ and postoperative factors play equally important roles in determining the course and severity of AKI; as such, continued risk assessment throughout the peri‐ and postoperative periods is crucial for patients at risk of AKI.

#### Definition and diagnosis of AKI

*Recommendations:*

1. We recommend that AKI should be defined by the KDIGO criteria, including both sCr and UO criteria (not graded).
2. We recommend checking sCr immediately before surgery in all patients and utilizing sCr‐based eGFR to assess renal function in patients with steady state preoperatively so as to ascertain AKI postoperatively (not graded).
3. We recommend repeated clinical reassessment of AKI risk within the first 12 postoperative hours incorporating intra‐ and postoperative variables (not graded).
4. We suggest measuring biomarkers of AKI (eg, TIMP2·IGFBP7 [combination of tissue inhibitor of metalloproteinases 2 and insulin‐like growth factor binding protein 7] or NGAL [neutrophil gelatinase–associated lipocalin]) in patients at high risk of CS‐AKI (grade 2A).

*Rationale:* The current Society of Thoracic Surgeons (STS) database defines AKI by KDIGO sCr‐based stage 3 AKI (sCr ≥3 times baseline or initiation of RRT).^74^ However, smaller changes in sCr are associated with adverse outcomes following CVS.^75^, ^76^, ^77^, ^78^ Given the association of stage 1 AKI (sCr 1.5 times baseline or ≥0.3‐mg/dL increase within 48 hours) with adverse outcomes in multiple settings, it is important to recognize stage 1 AKI (based on sCr and/or UO criteria) so that the progression to stage 2 (sCr 2.0 times baseline) or stage 3 AKI and other outcomes can be monitored. In addition, sCr criteria alone may miss ≈30% of patients with AKI, resulting in both misclassification of AKI severity and delay in management. Critically ill patients who meet AKI criteria by both sCr and UO are at higher risk of adverse outcomes including 30‐day mortality and need for RRT in comparison to those who meet a single criterion for AKI.^5^, ^79^, ^80^, ^81^ The STS database does not distinguish between AKI and acute kidney disease, which is currently defined as the course of the AKI syndrome in those who continue to have renal pathophysiological changes 7 days after the inciting event. Acute kidney disease may last for weeks to months with variable outcomes (full or partial recovery, ESRD).^82^ Moreover, the timing and trajectory of AKI (<7 days versus 7–30 days) after CVS can provide insight into the cause of kidney dysfunction and may likely have different associations with adverse outcomes.

We recommend checking sCr in all patients to determine preoperative baseline kidney function. The time frame depends on the presence of acute illness and other factors that may affect kidney health (eg, recent exposure to iodinated contrast in the setting of cardiac catheterization, medications including angiotensin‐converting enzyme inhibitors and angiotensin receptor blockers). In the absence of such factors, it is reasonable to obtain preoperative baseline sCr within 2 weeks of surgery. When sCr is at steady state (eg, stable for 48 hours), it can be used to calculate eGFR using validated estimating equations (eg, Chronic Kidney Disease–Epidemiology Collaboration [CKD‐EPI] or Modification of Diet in Renal Disease [MDRD]) to assess for CKD.^83^ For urgent or emergent surgical procedures, when sCr is less likely to be in steady state, the preoperative baseline sCr should be obtained within 24 hours of surgery and, ideally, as close as possible to the start of surgery. In these cases, clinical factors that contribute to the urgency of surgery (eg, cardiogenic shock) are likely to affect sCr and kidney function adversely.

##### Pitfalls of AKI definitions

Several specific challenges for AKI definitions exist in the context of cardiac surgery. In the setting of CPB, hemodilution frequently results in postoperative sCr below the preoperative baseline. Given the limitations of sCr, certain subpopulations may be particularly prone to overestimation of baseline kidney function, including elderly patients, sarcopenic patients, or those with significant volume overload. Given the importance of underlying CKD as a risk factor for perioperative AKI, this is most relevant for preoperative risk assessment. In these patients, quantitation of kidney function with cystatin C or timed creatinine clearances or eGFR equations (eg, CKD‐EPI or MDRD) may be preferable approaches.^83^

##### Beyond sCr

**Figure 3:**
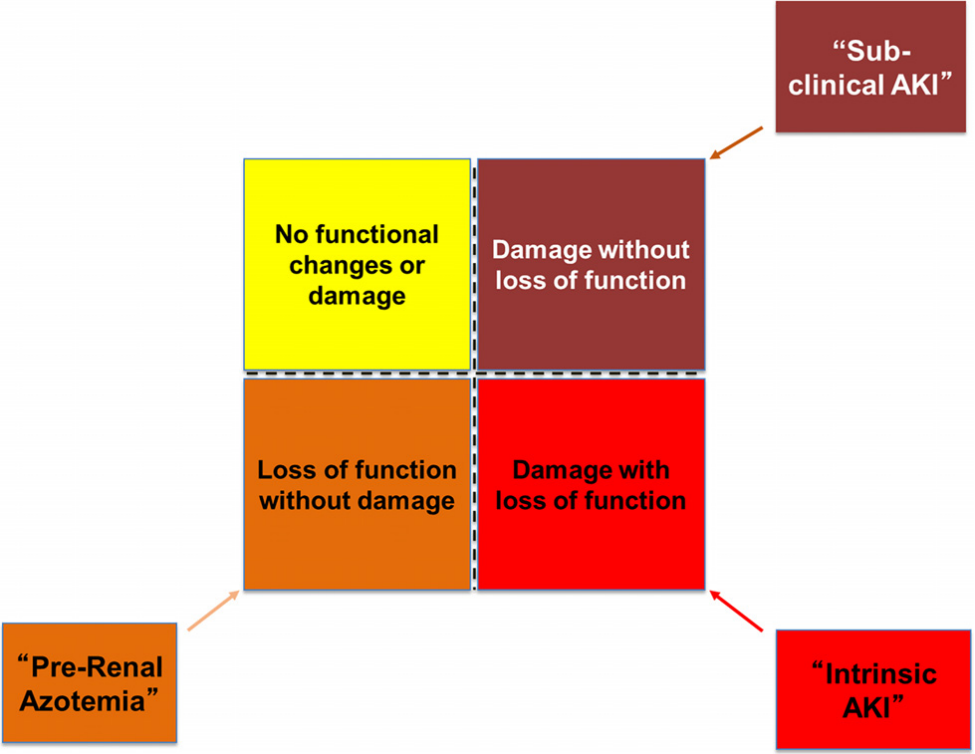
Classification of acute kidney injury (AKI) by changes in function and/or damage. Currently the diagnosis of AKI is made through changes in serum creatinine (sCr) or urine output (UO)—functional biomarkers. The 10th Acute Disease Quality Initiative consensus meeting delineated criteria for defining AKI in terms of changes in biomarkers of renal function (sCr/UO) and biomarkers of kidney damage. This paradigm allows for the combination of injury biomarkers with sCr and UO and has been useful in the discrimination of patients with AKI. The terms *prerenal* and *intrinsic* AKI are sometimes used to denote these relationships. The upper right box may be termed *subclinical*. The upper left box is yellow because we may still miss changes in function and damage in some patients; a role for additional diagnostics and/or stress tests is acknowledged.

Despite its ubiquitous use, sCr is an imperfect marker for the early detection of AKI because rises in sCr are delayed following the kidney insult. In patients with normal preoperative renal function, GFR may decrease significantly with only minimal effect on sCr. During cardiac surgery, CPB and intravenous fluid administration may lead to hemodilution of sCr in the perioperative period, delaying the recognition of AKI. These limitations of sCr (and other functional biomarkers of AKI such as UO) have led to a call for the use of tubular damage and stress biomarkers for the recognition and diagnosis of AKI.^82^ A recent consensus conference provided a framework for the reclassification of AKI along changes in functional (sCr and UO) and damage or stress biomarkers (Figure 3).^84^ Over the past decade, several biomarkers of AKI have demonstrated the ability to detect kidney injury or stress and have been proposed as tools to improve the evaluation and treatment of patients with AKI.^67^, ^85^, ^86^, ^87^, ^88^, ^89^, ^90^, ^91^, ^92^

Importantly, damage may occur before reaching the AKI threshold of changes in sCr or UO, reflecting delay in the kinetics of these functional markers or the presence of renal reserve (the compensatory ability of the kidney to increase GFR in the face of increased demand). The best‐studied and most widely available biomarkers in the clinical setting are urinary or plasma NGAL and TIMP2·IGFBP7.^67^, ^85^, ^86^, ^87^, ^88^, ^89^, ^90^, ^91^, ^92^, ^93^ NGAL has been most successful in pediatric cardiac surgery, whereas TIMP2·IGFBP7 has been used in both adult and pediatric cases. Thus, biomarkers may potentially refine the clinical management of AKI by providing both diagnostic and prognostic information independent of the conventional functional markers of AKI. Recent studies have demonstrated that implementing kidney‐focused care in the setting of postoperative patients with elevated stress biomarkers leads to improved patient outcomes (less AKI, less severe AKI, and decreased length of stay).^67^, ^93^ However, we recommend applying the KDIGO bundle only to CVS patients who are at high risk of AKI identified by biomarkers.

### Prevention of CVS‐AKI

*Recommendations:*

1. We recommend that a variety pharmacological and nonpharmacological strategies be used to prevent AKI in patients undergoing CVS (Table 1; grades 1A–2D).
2. We recommend avoiding certain interventions for prevention of AKI that have been shown to be ineffective or to cause harm (Table 1; grades 1A–1B).
3. A number of approaches to prevent AKI lack sufficient evidence for recommendation and are best avoided until such evidence is available (Table 1; not graded).
4. We recommend avoiding hydroxyethyl starch (grade 1A) and suggest judicious use of balanced crystalloid solutions rather than saline or albumin solutions to replace fluid losses (grade 2B).

**Table 1:** Strategies for Prevention of AKI After CVS

Timing	Strategy	Population	Recommended	Not Recommended
Perioperative	Avoidance of glucose variability	Cardiac	1B	…
	Balanced crystalloid (vs saline)	Cardiac and vascular	1B	…
	Dexmedetomidine	Cardiac	2C	…
	Statins	Cardiac	…	1A
	N‐acetylcysteine	Cardiac	…	1A
	Sodium bicarbonate	Cardiac	…	1A
	Levosimendan	Cardiac	…	1A
	Limited use of blood transfusion	Cardiac vascular	1A	More research needed
	Albumin (vs crystalloid)	Cardiac	…	More research needed
	Erythropoietin	Cardiac and vascular	…	More research needed
Preoperative	Discontinuation of ACEIs and ARBs	Cardiac	1C	…
	Albumin in patients with hypoalbuminemia	Cardiac (OPCAB)	2C	…
	24‐ to 72‐h delay postcontrast before cardiac surgery	Cardiac	2C	…
	IABP	Cardiac	2C^a^	…
Intraoperative	Volatile anesthetic agents (vs propofol)	Cardiac	2C	…
	Cold renal perfusion for AAA	Vascular	2C	…
	Avoidance of hyperthermia	Cardiac	2C	…
	Pulsatile CPB	Cardiac	2D^a^	…
	Avoidance of hemodilution	Cardiac	2C	…
	Techniques to prevent procedure‐related atheroembolism	Vascular	2C	…
	OPCAB technique	Cardiac	…	1A
	Remote ischemic preconditioning	Cardiac	2B^a^	More research needed for vascular and low risk cardiac surgery
	Minimization of aortic manipulation	Cardiac	…	More research needed
	MAP >75	Cardiac	…	More research needed
	Intraoperative ultrafiltration	Cardiac	…	More research needed
Postoperative	KDIGO bundle	Cardiac	1B^a^	…
	Low tidal volume ventilation strategy	Cardiac	1C	…
	Loop diuretics (for prevention of AKI)	Cardiac and vascular	…	1B
	Levosimendan	Cardiac	…	1A
	Dopamine	Cardiac	…	1A
	A‐melanocyte‐stimulating hormone	Cardiac	…	1B
	Vasopressin for vasoplegic shock (vs norepinephrine)	Cardiac	…	More research needed
	Natriuretic peptides	Cardiac	…	More research needed
	Fenoldopam	Cardiac	…	More research needed
	Mannitol	Cardiac and vascular	…	More research needed

AAA indicates abdominal aortic aneurysm (includes thoracoabdominal); ACEI, angiotensin‐converting enzyme inhibitor; AKI, acute kidney injury; ARB, angiotensin receptor blocker; CPB, cardiopulmonary bypass; CVS, cardiac and vascular surgery; IABP, intra‐aortic balloon pump; KDIGO, Kidney Disease: Improving Global Outcomes; MAP, mean arterial pressure; OPCAB. off‐pump coronary artery bypass.

a High‐risk cardiac surgery patients.

*Rationale:* The evidence for most pharmacological and nonpharmacological strategies in the perioperative setting is limited because the majority of studies are single center, are of poor quality with small sample sizes, and/or use variable inclusion criteria. In addition, the timing and dose of pharmacological agents and the definition of AKI vary widely.

#### Pharmacological strategies

##### Perioperative

Numerous pharmacological agents including levosimendan,^94^, ^95^, ^96^, ^97^ statins,^98^, ^99^, ^100^ N‐acetylcysteine,^101^, ^102^, ^103^, ^104^ sodium bicarbonate,^105^, ^106^, ^107^, ^108^ and erythropoietin^109^, ^110^, ^111^, ^112^, ^113^ have, for the most part, failed to demonstrate benefit for the prevention of CS‐AKI. A possible exception is dexmedetomidine, for which a number of small or low‐quality studies found a reduction in the occurrence of AKI after cardiac surgery.^114^, ^115^, ^116^ Moderate glucose control (127–179 mg/dL) was found in a randomized controlled trial (RCT) to be preferable to tight control (≤126 mg/dL) in patients undergoing coronary artery bypass grafting,^117^ resulting in lower rates of AKI and mortality, with the most important factor being avoidance of glucose variability throughout the entire perioperative time frame.^118^, ^119^ The use of balanced crystalloid solutions guided by measures of fluid responsiveness is supported by the current literature and is consistent with the recommendations for fluid management in critically ill patients with sepsis.^97^, ^120^, ^121^, ^122^, ^123^ The administration of hydroxyethyl starch is not indicated for patients at risk of CS‐AKI because of its demonstrated renal toxicity.^97^, ^121^ Although albumin may have a role preoperatively in patients with hypoalbuminemia,^124^ we suggest limiting colloid administration in cardiac surgery patients as much as possible and recommend balanced crystalloid solutions as replacement fluid, in keeping with the literature.^120^

##### Preoperative

Although evidence is limited, several studies demonstrate that withholding angiotensin‐converting enzyme inhibitors and angiotensin receptor blockers in the preoperative period is associated with reduced incidence of AKI.^125^, ^126^, ^127^ Correction of hypoalbuminemia (level of <4 g/dL) by exogenous albumin supplementation has been shown to be renoprotective in off‐pump cardiac surgery.^124^, ^128^

##### Intraoperative

Volatile anesthetics have been shown to protect against AKI in clinical trials.^129^, ^130^ A recently published meta‐analysis including 10 trials with 1600 total participants demonstrated that volatile anesthetics significantly reduced AKI incidence compared with control data (relative risk: 0.65; 95% confidence interval, 0.43–0.97; *P*=0.04). Although there was no significant difference between groups in absolute postoperative sCr level and mortality, patients receiving volatile anesthetics had significantly (or borderline) lower increase in sCr level from baseline on the first and second postoperative days and reduced incidence of prolonged intensive care unit stay and hospitalization.^131^

##### Postoperative

For agents such as dopamine,^132^ α‐melanocyte–stimulating hormone analog,^133^ and loop diuretics,^134^, ^135^ evidence shows that their use in the prevention of AKI either is not beneficial or is potentially harmful.^132^, ^134^, ^135^ Because the evidence for mannitol^136^, ^137^, ^138^ and fenoldopam^139^, ^140^, ^141^, ^142^, ^143^ is either weak or controversial, more research is needed. Although natriuretic peptides have been shown in some studies to reduce the incidence of AKI, the need for RRT was similar, and the overall evidence for these drugs was weak and controversial.^144^, ^145^, ^146^, ^147^, ^148^, ^149^, ^150^, ^151^, ^152^ Vasodilatory shock (vasoplegia) has become a common occurrence, particularly with the widespread use of angiotensin‐converting enzyme inhibitors and angiotensin receptor blockers.^153^ In a recent RCT of patients with vasoplegic shock (defined as mean arterial pressure <65 mm Hg resistant to fluid challenge and cardiac index >2.2 L/min per m^2^) after cardiac surgery, vasopressin was associated with lower incidence of severe AKI (stages 2 and 3) and RRT use, although, again, study limitations prevent recommendation and further research is needed.^154^

#### Nonpharmacological strategies

##### Perioperative

Again, results in this area are contradictory. Intravenous contrast before surgery may increase the incidence of AKI and has led to some recommendations for delaying surgery 24 to 72 hours after contrast administration^155^, ^156^; however, another study disagrees.^157^ Preoperative placement of an intra‐aortic balloon pump may prevent AKI and reduce the incidence of RRT in high‐risk patients by improving perfusion and reducing endothelial activation.^158^, ^159^ Others, however, have suggested that using an intra‐aortic balloon pump as a surrogate to achieve pulsatile perfusion contributes significantly to lowered aortic pressure in the distal portion of aorta and may impair kidney perfusion.^160^

##### Intraoperative

The use of CPB is required for the majority of cardiac surgical procedures. During CPB, cardiac output is preserved, but the target blood pressure under such nonpulsatile conditions is unknown. A large RCT demonstrated that maintaining a higher level of mean arterial pressure during normothermic CPB did not reduce the incidence of AKI.^161^ However, before making any recommendations, further evidence is required. The impact of off‐pump coronary artery bypass (OPCAB) on renal outcomes has been studied extensively and remains another point of controversy. OPCAB exerted a renoprotective effect in patients with normal preoperative sCr but not in patients with preexisting renal disease.^162^ No impact on the incidence of AKI with the 2 competing strategies was seen in some series,^163^, ^164^ whereas others found an association of on‐pump coronary artery bypass with higher incidence of AKI.^165^ A meta‐analysis of trials (n=17 322 patients) suggests a lower risk for AKI but no difference in the need for RRT in off‐pump surgery patients.^166^ However, a recently published RCT (n=2932 patients) demonstrated that OPCAB reduced the risk of postoperative AKI in comparison to on‐pump coronary artery bypass grafting, with no discernable difference in kidney function at 1 year.^41^ Although data suggest a reduction in AKI in OPCAB, a recent RCT^42^ and several meta‐analyses^167^, ^168^ have demonstrated an increase in long‐term, all‐cause mortality in OPCAB compared with on‐pump surgery. Consequently, the greater operative safety and possible prevention of AKI with OPCAB may come at the expense of long‐term survival gains.

Catheter manipulation in the thoracic and abdominal aorta may lead to renal artery embolization, with aortic cross‐clamping proximal to the renal arteries associated with ischemia–reperfusion injury, further aggravated by atheroemboli secondary to aortic manipulation.^169^, ^170^ Aortic clamping above bilateral renal arteries, adjunctive renal artery procedures, and left renal vein division have been found to increase the incidence of AKI.^136^ Emboli protection devices may help preserve renal function and prevent procedure‐related atheroembolism during endovascular renal interventions.^171^ Transradial coronary angiography avoids catheter manipulation in the descending and abdominal aorta, which, compounded by reduced bleeding, leads to less AKI than transfemoral angiography.^172^ Avoidance of aortic manipulation (the “no touch” technique) in OPCAB has been shown to decrease the risk of postoperative stroke in several studies; however, its effect on AKI incidence remains controversial, with some studies demonstrating lower incidence,^173^, ^174^ but others failing to do so.^175^, ^176^

Moderate hypothermic circulatory arrest with antegrade cerebral perfusion during complex aortic surgery has been embraced by an increasing number of surgical groups, with data suggesting that this is not associated with an increased AKI risk.^177^ Conversely, hyperthermic perfusion during CPB, defined as a cumulative time at >37°C, is associated with an increase in AKI incidence.^178^ In a study achieving clinical equipoise through propensity score matching, duration of hyperthermic perfusion was independently associated with severity of AKI, with a 51% increase in the incidence for every 10 minutes of hyperthermic perfusion.^179^ Cold renal perfusion has been suggested for pararenal abdominal aorta aneurysm surgery to reduce the incidence of AKI associated with juxtarenal and thoracoabdominal aortic operations.^180^, ^181^, ^182^

Pulsatile perfusion provides surplus mechanical energy transmission to the vascular endothelium. Its impact on clinical outcomes has been extensively studied in a variety of settings; however, the data on renal outcomes are conflicting because of the lack of uniformity in pulsatility delivery.^159^, ^183^, ^184^, ^185^ A retrospective analysis showed pulsatile CPB conferred a renoprotective effect in higher risk patients undergoing cardiac surgery.^186^ However, the contemporary use of implantable continuous‐flow left ventricular assist devices challenges this concept.^187^ The improvement in the hemodynamic environment provided by continuous‐flow left ventricular assist devices leads to better renal function in heart failure patients, implying that the theoretical importance of pulsatility is superseded by increased cardiac output.^187^, ^188^

Hemodilution during CPB is an independent risk factor for AKI in adult cardiac surgery,^189^ with improved outcomes for cases in which significant hemodilution (hematocrit <24%) is avoided during CPB.^190^ Although lower hemoglobin (8.8 versus 13.1 g/dL) preoperatively and on arrival to the intensive care unit has been associated with persistent AKI after cardiac surgery,^191^ transfusion of at least 2 U of packed red blood cells has also been associated with higher incidence of CVS‐AKI.^192^ In 2 recent RCTs in which patients were randomized to a liberal (Hg <9.5 g/dL) or restrictive (Hg <7.5 g/dL) transfusion policy intraoperatively and postoperatively, there was no difference in postoperative outcomes including AKI.^193^, ^194^ In addition, studies have failed to demonstrate any benefit in the use of erythropoietin in the prevention of postoperative AKI.^109^, ^110^, ^111^ Conventional ultrafiltration has been used to treat the hemodilutional effects of CPB circuits, but routine adoption does not seem to be efficacious, with a recent study demonstrating a higher adjusted risk of AKI.^195^

As discussed, the effect of RIPC on renal function yields conflicting results.^24^, ^25^, ^26^ In high‐risk patients undergoing cardiac surgery, the rate of AKI and the need for RRT have been found to be significantly decreased using RIPC,^196^ whereas in studies of unselected patients with nonstandardized intraoperative technique, no renal benefit was observed.^25^, ^26^ Results of recent meta‐analyses have failed to demonstrate the efficacy of RIPC in reducing the incidence of AKI or the need for RRT.^197^, ^198^, ^199^, ^200^ Importantly, these meta‐analyses included studies involving low‐risk patients and studies that used propofol. In a subgroup of studies in which propofol was not used, a reduction in AKI was demonstrated, suggesting a possible interaction of propofol with the protective effects of RIPC.^200^ Data for vascular surgery patients are also conflicting and limited to 4 small RCTs in patients undergoing open or endovascular abdominal aortic aneurysm repair, the majority of whom showed no benefit with RIPC in preventing AKI.^201^, ^202^, ^203^, ^204^

##### Postoperative

Implementation of a “KDIGO bundle of care”—consisting of avoidance of nephrotoxic agents, discontinuation of angiotensin‐converting enzyme inhibitors and angiotensin receptor blockers for the first 48 hours after surgery, close monitoring of sCr and UO, avoidance of hyperglycemia for the first 72 hours after surgery, consideration of alternatives to radiocontrast agents, and close hemodynamic monitoring using a prespecified algorithm—prevented CVS‐AKI in high‐risk patients defined as biomarker positive.^67^, ^93^ A lower rate of CS‐AKI has been shown in patients ventilated with low (<10 mL/kg) tidal volume compared with traditional (10–12 mL/kg) or high (>12 mL/kg) tidal volumes after cardiac surgery.^205^

### Management of CVS‐AKI

#### Workup and monitoring

*Recommendations:*

1. The choice of diagnostic tests and monitoring for patients with CVS‐AKI is determined by the presence and the degree of kidney and/or cardiorespiratory dysfunction (not graded).
2. The diagnostic approach depends on the etiology of organ dysfunction and the expected short‐ and long‐term patient outcomes (not graded).
3. A kidney‐specific and stepwise diagnostic approach for patients with CVS‐AKI and cardiorespiratory dysfunction may be helpful (not graded).

**Figure 4:**
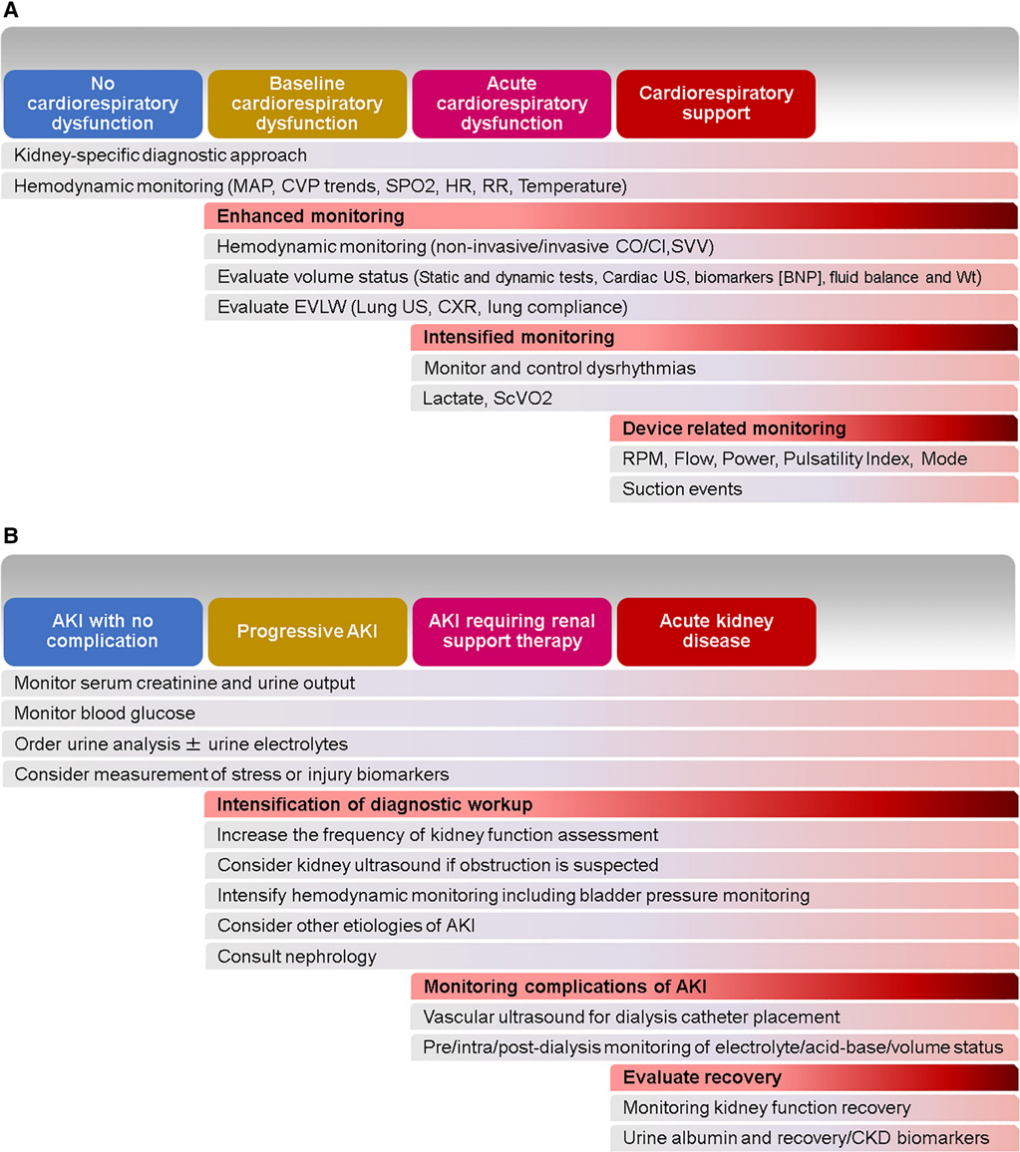
A, Cardiorespiratory‐specific diagnostic approach. This diagnostic approach may be applied to a patient who has a cardiorespiratory cause of acute kidney injury (AKI). The level of intervention is governed by the degree and chronicity of cardiorespiratory dysfunction. Source: ADQI (Acute Disease Quality Initiative) 20th consensus meeting (http://www.adqi.org). Used with permission. B, Kidney‐specific diagnostic approach. This diagnostic approach may be applied to a patient who has a renal‐specific cause of AKI. The level of intervention is governed by the degree and duration of renal dysfunction. This is particularly relevant in the post–intensive care unit phase, in which a patient with persistent AKI (>2 or 3 days) or acute kidney disease should be monitored and followed up. BNP indicates brain natriuretic peptide; CI, cardiac index; CKD, chronic kidney disease; CO, cardiac output; CVP, central venous pressure; CXR, chest x‐ray; EVLW, extravascular lung water; HR, heart rate; MAP, mean arterial pressure; RR, respiratory rate; Scvo_2_, central venous oxygen saturation; Spo_2_, peripheral oxygen saturation; SVV, stroke volume variation; US, ultrasound.

*Rationale:* Effective interventions to reverse the consequences of AKI remain elusive, with efforts focusing on primary prevention. Nevertheless, management of patients who sustain AKI is of vital importance, both to reduce the immediate impact of AKI and to avoid further episodes of AKI and avert the progression of AKI to acute kidney disease or CKD. Organ functional changes are dynamic, and, as such, frequent assessments are required to establish the trajectory that will influence the nature and urgency of interventions. Such assessments should guide preemptive therapies to prevent or manage complications that are anticipated in the setting of AKI. Because the impact of CVS‐AKI on both short‐ and long‐term outcomes of patients is significant, reaching appropriate diagnoses to provide etiology‐specific individualized preventive and/or therapeutic measures is critical. The diagnostic tests and monitoring strategies available are manifold (Table 2) and should be determined by the level of kidney or cardiorespiratory dysfunction present (Figure 4A and 4B).

**Table 2:** Suggested Diagnostic Tests and Monitoring Strategies for CVS‐AKI

Category	Test	Patients	Advantage	Disadvantage
Cardiovascular function	Basic hemodynamic monitoring (MAP, cardiac filling pressure, and its trends, Spo_2_, HR, RR, temperature)	All patients	Identification of patients with need for advanced monitoring Ability to evaluate the trends	Low resolution Questionable relationship with volume deficiency
	Advanced hemodynamic monitoring (thermodilution, transpulmonary indicator dilution, arterial‐pressure waveform‐derived, esophageal or suprasternal Doppler, echocardiography, partial CO_2_ rebreathing, bioimpedance, and bioreactance)	Progressive or severe AKI Hemodynamic instability	Accuracy High resolution	Invasive Limited evidence for effect on outcomes
	Assist device–related information	Patients who need cardiorespiratory support	Readily available Informative	Requires expertise to interpret
	Lactate	Progressive or severe AKI Hemodynamic instability	Indicator of tissue O_2_ delivery Clearance guides therapy	Lacks specificity (eg, epinephrine‐induced increase in lactate)
	Svo_2_ or Scvo_2_	Progressive or severe AKI Hemodynamic instability	Balance of tissue O_2_ delivery and consumption	Sensitivity can be reduced with shunting and when O_2_ consumption is low
Pulmonary function	Blood gas	Cardiorespiratory dysfunction	Accurate measure of oxygenation and ventilation	Limited information concerning cardiac performance
	Lung US	Cardiorespiratory dysfunction	Noninvasive Sensitive and specific	Interrater variability
	Lung imaging	Cardiorespiratory dysfunction	Simple, uniformly available	X‐ray exposure; Specificity can be limited
	Lung compliance	Patients on MV	Easy to perform	May lack specificity
Kidney function	sCr	All patients with CVS‐AKI	Available Informative (diagnosis and prediction)	Late and insensitive biomarker of kidney dysfunction
	Urine sediments	Kidney dysfunction	High specificity	Low sensitivity
	Urine electrolytes	Kidney dysfunction	Available Confirmatory	Limited sensitivity and specificity, Confounded by diuretics
	Injury/stress biomarkers	CVS‐AKI (AKI risk and stage I)	Informative (diagnosis, prognosis) Less delayed	Limited availability in some areas
	Kidney US	Suspicion of obstruction	Highly sensitive and specific	Interrater variability
	Kidney Doppler US	Progressive kidney dysfunction	Noninvasive	Lack of specificity of resistive index Limited images in ICU
	Urine eosinophils	Progressive kidney dysfunction and clinical suspicion of AIN/atheroembolic disease	Reasonable specificity	Low sensitivity
	Kidney biopsy	Progressive kidney disease with unknown etiology	Informative (diagnosis, prognosis)	Not done in usual clinical practice
Miscellaneous	Chemistry panel	All patients with CVS‐AKI	Available Informative (eg, to assess AKI complications)	
	Glucose monitoring	All CVS‐AKI patients	Allows appropriate glucose management	
	Myoglobin and sarcoplasmic proteins (eg, creatine kinase, aldolase, LDH, ALT, and AST)	Progressive AKI with clinical suspicion	Informative (rhabdomyolysis diagnosis)	
	Complement	Progressive AKI with clinical suspicion of immunologically mediated renal disease (eg, atheroembolic disease, infection, I/R)	Confirmatory	Low sensitivity
	ESR/CRP	Progressive AKI with clinical suspicion (eg, Atheroembolic disease, infection, I/R)	Confirmatory	
	Peripheral blood smear and hemolysis panel	Progressive AKI with clinical suspicion of hemolysis associated AKI (eg, HUS/TTP, DIC)	Confirmatory	
	Inflammatory biomarkers	Vasodilatory shock	Predictor of outcome	Limited availability; no standardization

AIN indicates acute interstitial nephritits; AKI, acute kidney injury; ALT, alanine aminotransferase; AST, aspartate aminotransferase; CRP, C‐reactive protein; CVS, cardiac and vascular surgery; DIC, disseminated intravascular coagulation; ESR, erythrocyte sedimentation rate; HR, heart rate; HUS, hemolytic uremic syndrome; ICU, intensive care unit; I/R, ischemia/reperfusion; LDH, lactate dehydrogenase; MAP, mean arterial pressure; MV, mechanical ventilation; RR, respiratory rate; sCr, serum creatinine; Spo_2_, peripheral oxygen saturation; Scvo_2_, central venous oxygen saturation; Svo_2_, systemic venous oxygen saturation; TTP, thrombotic thrombocytopenic purpura; US, ultrasound.

If no evidence shows cardiopulmonary failure, it is essential to try to identify the etiology of the AKI. Following evaluation of cardiorespiratory function and intravascular volume status, urine analysis and medication review are initial steps in assessing such patients. The use of stress or injury biomarkers of AKI may potentially allow more accurate prognostication of AKI because they have been shown to correlate with long‐term outcomes.^206^, ^207^ When AKI persists for >48 to 72 hours, nephrology consultation to evaluate other potential causes of AKI and, potentially, disease‐specific management may be helpful. For patients who require RRT, close monitoring of metabolic milieu and hemodynamic/volume status is critical. Once discharged, clinicians should follow patients who recovered from CVS‐AKI for the development of CKD or other post‐AKI complications.^2^

#### Pharmacological and nonpharmacological support

*Recommendations:*

1. The management goals for the CVS patient who has sustained AKI include preventing the progression of AKI, promoting renal recovery precluding subsequent episodes of AKI, and treating the acute and chronic consequences of AKI (not graded).
2. We recommend not using natriuretic peptide, fenoldopam, diuretics, dopamine, or mannitol for the treatment of CVS‐AKI (grade 1C).
3. The decision to start RRT should be individualized with consideration of the clinical context and not be based solely on renal function or stage of AKI. Once the decision to initiate RRT has been made, it should be started promptly (not graded).
4. We recommend the use of continuous therapies in patients with hemodynamic instability and in situations in which shifts in fluid balance are poorly tolerated (grade 1B).

*Rationale:* For those patients with CVS‐AKI who present with cardiorespiratory failure, the diagnostic approach needs to focus first on addressing cardiorespiratory dysfunction. Among patients who have significant baseline or acute cardiorespiratory failure or who require substantial support for heart and lung function, the intensity of hemodynamic and intravascular volume status monitoring should be escalated.^208^ The hemodynamic management of cardiac surgery patients with renal dysfunction should focus on:

1. Improvement of right and left ventricular function
2. Maintenance of MAP and sinus rhythm
3. Optimization of preload
4. Management of right ventricle afterload (pulmonary vascular resistance)
5. Optimization of mechanical ventilation

##### Pharmacological cardiac support

A multipharmacological approach is frequently necessary. Catecholamines are often required to improve ventricular function, acting through a direct inotropic effect but also by improving myocardial perfusion and recruitment of unstressed venous preload. Phosphodiesterase type III inhibitors (milrinone and enoximone), calcium sensitizers (levosimendan), and pulmonary vasodilators (inhaled nitric oxide, inhaled prostacyclin, and inhaled milrinone) can improve cardiac performance while reducing afterload on both right and left ventricles. Because the determinant for renal perfusion is the arterial and venous blood pressure, both left ventricle dysfunction and increased central venous pressure are associated with decreased renal perfusion and increased renal afterload contributing to CS‐AKI.^209^ As noted, increased renal venous pressure also causes an increase in the renal subcapsular pressure, thereby reducing glomerular filtration.^210^ The use of dynamic measures based on heart–lung interactions to predict fluid responsiveness, such as pulse pressure or stroke volume variation, has been shown to improve outcomes including renal function in both cardiac and noncardiac settings.^211^ Fluid overload is particularly problematic following cardiac surgery because of the frequent co‐occurrence of low cardiac output and postcardiotomy ventricular impaired relaxation. Following the acute phase, slow and controlled volume unloading, through the use of diuretics and/or continuous RRT, is frequently necessary.

##### Pharmacological kidney support

Pharmacological management of CVS‐AKI remains challenging, with no specific approaches available currently. Many drugs including dopamine, loop diuretics, mannitol, and natriuretic peptides have been investigated as primary renal therapy. Although associated with increased UO, none are routinely used because of limited and conflicting data and, in some cases, evidence of harm.^140^, ^145^, ^212^ RCTs with natriuretic peptides have shown inconsistent effects for renal end points. Meta‐analyses demonstrated that atrial natriuretic peptide in high doses was associated with a trend toward increased mortality and more adverse events in patients undergoing cardiac surgery.^144^ Although there was a reduced need for RRT (with the associated problems using RRT as an end point) with low‐dose atrial natriuretic peptide in patients undergoing CVS, there was no difference in mortality. The majority of studies on atrial natriuretic peptide are underpowered and of low or moderate quality; therefore, atrial natriuretic peptide is not currently recommended for treatment of AKI.^213^ Although meta‐analyses have suggested a decrease in RRT in patients with CS‐AKI who were treated with fenoldopam, a multicenter, randomized, double‐blind, placebo‐controlled, parallel‐group study of patients with CS‐AKI (n=667) was stopped for futility after an interim analysis. Fenoldopam infusion did not reduce 30‐day mortality or the need for RRT but was associated with an increased rate of hypotension compared with placebo.^140^

##### Renal replacement therapy

**Figure 5:**
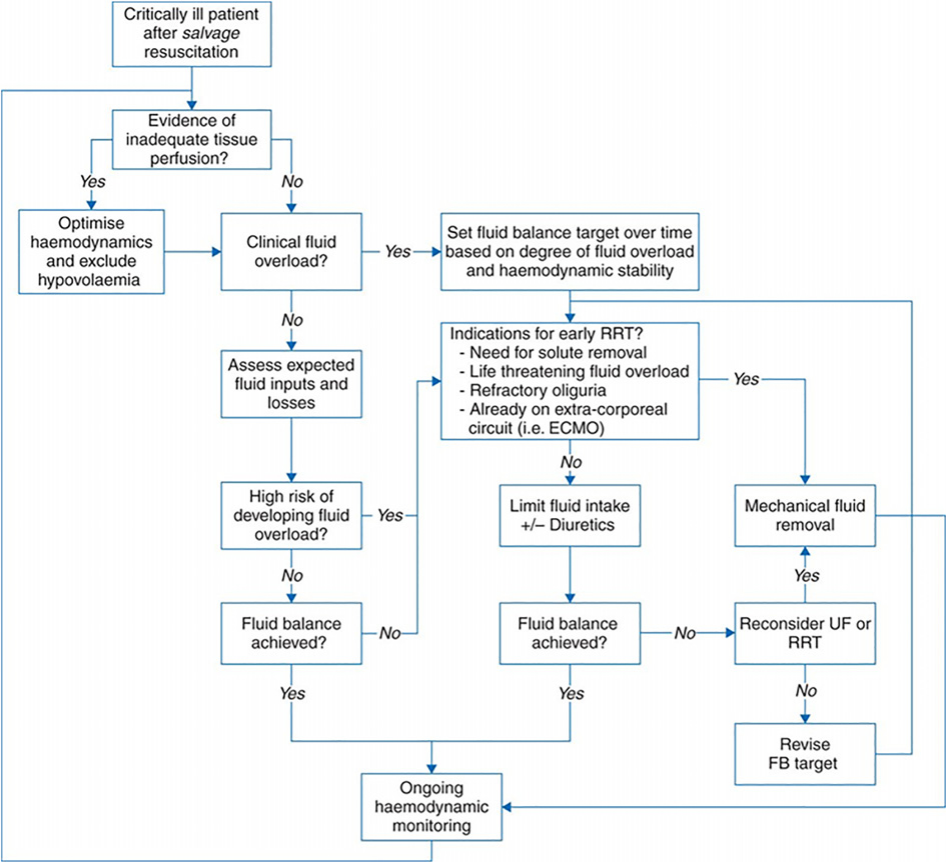
Fluid management strategies in critical illness: the place of mechanical fluid removal. Once life‐threatening hypovolemia has been corrected (savage resuscitation), fluid overload (FO) needs to be avoided. Early mechanical fluid removal should be considered if specific indications exist. Note, the existence of an extracorporeal circuit for extracorporeal membrane oxygenation (ECMO) greatly reduces any added risk for renal replacement therapy (RRT), assuming this circuit is used rather than a separate line for RRT. However, some patients will respond well to diuretics, and thus an ECMO circuit in place is only a relative indication for early RRT initiation and only when fluid or solute management dictates. During therapy, hemodynamic and intravascular volume status should be monitored and fluid removal rate and fluid balance targets reassessed regularly, aiming for clinical stability and tolerance of fluid removal. Within this pathway, RRT should be considered at any point if additional solute clearance is necessary. FB indicates fluid balance; UF, ultrafiltration.

In patients undergoing cardiac surgery, perioperative fluid overload has been associated with worse outcomes and is a primary risk factor for multiorgan failure, including AKI.^214^, ^215^, ^216^ Fluid balance can be achieved with pharmacological agents; however, in some cases, RRT should be considered to correct fluid accumulation, even in nonoliguric patients if the daily fluid balance cannot be maintained or is negative with the use of diuretics, to avoid the negative effects of prolonged fluid overload in critically ill patients (Figure 5).^217^, ^218^, ^219^

The optimal timing of RRT initiation remains a topic of much debate. Theoretically, initiation of RRT before the onset of severe AKI could improve survival and promote earlier recovery of kidney function by mitigating injury from acidemia, uremia, fluid overload, and systemic inflammation.^220^ However, early initiation may put patients at risk associated with RRT when a conservative approach could be used. Meta‐analyses examining the timing of initiation of RRT^221^, ^222^, ^223^, ^224^ have suggested that earlier initiation of RRT in critically ill patients with AKI may have a beneficial impact on survival. A few small RCTs were included, as well as numerous observational and cohort studies of various qualities with high heterogeneity. Although AKI stage correlates with hospital mortality, many patients with stage 3 AKI will show spontaneous recovery without RRT. In addition, some patients may have urgent indications for RRT (eg, fluid overload not responding to diuretics) without meeting severe AKI criteria. The decision to start RRT should be individualized.^219^ Recently, 2 large RCTs in critically ill patients compared “early” versus “late” RRT.^225^, ^226^ In the ELAIN (Early vs Late Initiation of Renal Replacement Therapy In Critically Ill Patients With Acute Kidney Injury) trial (n=231), patients with AKI stage 2 and serum NGAL >150 ng/mL were randomized to early (RRT within 8 hours of AKI stage 2) or late (RRT within 12 hours of reaching AKI stage 3) RRT.^225^ Approximately half of the patients were cardiac surgery patients (47%). Patients in the early RRT group had 25% lower mortality compared with those in the late arm. Only 10% of patients in the late group did not receive RRT. In the AKIKI (Artificial Kidney Initiation in Kidney Injury) trial (n=620), patients were randomized to early treatment, defined as RRT within 6 hours of reaching AKI stage 3, or late treatment, defined as RRT initiation when “absolute” indications were fulfilled.^226^ Mortality (≈50%) was not significantly different between the 2 groups, but approximately half of the patients randomized to late treatment did not receive RRT. Patients in the late treatment group who started RRT had higher mortality (62%) compared with patients in the early RRT group (48.5%). Forty percent of the patients were excluded on the basis of having emergent indications, already receiving RRT, or having indications for initiation of RRT for >5 hours. Clearly, these 2 trials do not definitively identify which patients are likely to benefit most from early initiation of RRT.

Studies of dialysis modality (continuous versus intermittent) have failed to demonstrate a consistent benefit of one technique over another.^227^ However, continuous RRT is recommended for patients with hemodynamic instability that exceeds the ability to manage patients safely with intermittent hemodialysis.^213^, ^219^, ^228^ In addition, fluid removal is generally easier and associated with less hemodynamic instability when using continuous RRT.^229^, ^230^ It is possible that the operational characteristics of RRT might influence renal and patient recovery. Several observational studies have suggested that intermittent hemodialysis compared with continuous RRT is associated with less renal recovery and a higher risk of long‐term dialysis dependency.^231^, ^232^

##### Stem cells

Use of mesenchymal stem cells, which are nonhematopoietic precursor cells derived from human bone marrow, is currently under investigation (2 trials completed and being analyzed) for the treatment of CS‐AKI.^233^, ^234^ The role of mesenchymal stem cells in AKI treatment might depend on the secretion of promitotic, antiapoptotic, anti‐inflammatory, and immunomodulatory factors.^235^

## Knowledge Gaps and Future Research

Currently, there is a paucity of data regarding diagnosis, prevention, and treatment, especially in patients undergoing vascular surgery. Consequently, it is imperative to develop research questions to address these issues (Table 3). For many of the pharmacological agents available for the prevention or management of AKI in this setting, larger RCTs will be needed to determine efficacy in different clinical scenarios. It is important to establish the utility of new biomarkers, not only for the early diagnosis and staging of AKI but also for the implementation of preventive measures.

**Table 3:** Knowledge Gaps and Future Research Directions in CVS‐AKI

Knowledge Gap	Future Research Directions
Risk assessment	Defining the association of KDIGO stage 1 AKI by urine output and sCr with outcomes in the CVS settings Investigation of acute kidney stress are warranted to better characterize the incidence and outcomes of those with elevations in injury and damage biomarkers before changes in sCr and urine output Development of iterative risk‐prediction models that allow reevaluation of risk in the pre‐, peri‐, and postoperative periods. In this context, we recommend evaluation of the incremental value of real‐time estimated GFR assessment and renal injury/stress biomarkers as part of a risk stratification strategy Feasibility studies to assess renal reserve in the preoperative period for unrecognized renal susceptibility in selected group of patients
Risk stratification	Development and validation of current and emerging biomarkers of AKI diagnosis, recovery, progression to CKD Research and development of noninvasive, inexpensive, and highly accurate devices for kidney function, hemodynamic, and volume status monitoring (eg, real‐time GFR monitoring devices, kidney perfusion, and intracapsular pressure monitors, etc) Design and investigation of the impact of the biomarker or technology‐guided protocols in the prevention or treatment of CVS‐AKI
Prevention of CVS‐AKI	Development of biomarker or diagnostic tool‐guided protocols to prevent the progression of CVS‐AKI or facilitate kidney function recovery Investigation and validation of biomarkers and diagnostic tools with more resolution or ability to identify intravascular volume deficiency, microcirculation deficits, and kidney‐related variables and outcomes (eg, severity and location of injury, real‐time kidney function measures, biomarkers of kidney recovery, fibrosis or de novo or progression of CKD or need for RRT) Development of noninvasive, inexpensive, and highly accurate devices for kidney function, hemodynamic, and volume status monitoring (eg, real‐time GFR monitoring devices, kidney perfusion, and intracapsular pressure monitors, etc)
Management of CVS‐AKI	Development of studies to verify if specific management approaches currently showed as effective in CVS‐AKI primary prevention are also useful for secondary prevention Improvement of definition and monitoring of fluid overload in order to better understand its relationship and management strategies in CVS‐AKI patients Development of large randomized trial on ANP for the prevention and treatment of AKI Evaluation of the role of stem cells in treatment of AKI

AKI indicates acute kidney injury; ANP, atrial natriuretic peptide; CKD, chronic kidney disease; CVS, cardiac and vascular surgery; GFR, glomerular filtration rate; KDIGO, Kidney Disease Improving Global Outcome; RRT, renal replacement therapy; sCr, serum creatinine.

## Sources of Funding

This conference was supported by educational grants from OneLegacy Foundation, Baxter, NxStage Medical, Astute Medical, Sphingotec, Fresenius, Ortho Clinical Diagnostics, AM‐Pharma and La Jolla Pharmaceutical.

## Disclosures

Nadim has acted as a consultant for Sphingotec and Baxter. Forni has received speakers fees from Astute medical and as a consultant for Ortho Clinical Diagnostics, Baxter and La Jolla Pharmaceuticals. Bihorac has received speakers honoraria from Astute Medical. Koyner has received research funds from Astute Medical, Bioporto, Satellite Health Care, speakers honoraria from the American Society of Nephrology, expert witness on AKI post cardiac surgery, and consultancy work for Sphingotec and Pfizer. Shaw has received consultancy fees from Edwards, Astute Medical, FAST Biomedical and NxStage. Engelman has received consultancy fees from Astute Medical, Astellas. Liu has received consultancy fees from Potrero Medical, Quark and Theravance. Mehta has received consultancy fees from Baxter, Astute Medical, Sphingotec. Pickkers, Radboud University Medical Center, has received consultancy fees from Baxter, Sphingotec and AM Pharma. Zarbock, has received research fees from Astute Fresenius, Astute Medical, Fresenius, Astellas, Baxter and consultancy fees from Sphingotec and Astellas. Kellum, has received research funding from, Astellas Baxter Bioporto Astute Medical, Grifols Cytosorbents Bard, NxStage, and has received consultancy fees from, Astellas, Baxter, Bioporto, Grifols, Astute Medical, NxStage, Cytosorbents, Sphingotec.

## Supporting information


**Table S1.** Information Regarding Workgroups and Work Product
**Data S1.** ADQI (Acute Disease Quality Initiative) methodology.
