# Supplementary material for: Cardiac and Vascular Surgery–Associated Acute Kidney Injury: The 20th International Consensus Conference of the ADQI (Acute Disease Quality Initiative) Group
Source: J Am Heart Assoc. 2018 Jun 1;7(11):e008834. doi: 10.1161/JAHA.118.008834 (PMC6015369; doi:10.1161/JAHA.118.008834)
Supplement: Supplementary file 1 — Table S1. Information Regarding Workgroups and Work Product Data S1. ADQI (Acute Disease Quality Initiative) methodology. [file JAH3-7-e008834-s001.pdf]

SUPPLEMENTAL MATERIAL

Appendix

Information regarding workgroups and work product

Co-Chairs	Group 1	Group 2	Group 3	Group 4
**Mitra K. Nadim (Los Angles, CA, USA)**<br><br>**Lui G. Forni (Guildford, UK)**<br><br>**John Kellum (Pittsburgh, PA, USA)**<br><br>**Claudio Ronco (Vicenza, Italy)**<br><br>**Vladimir Gasparovic (Zagreb, Croatia)**	Risk Assessment for the development of CVS-AKI	Pathophysiology of CVS-AKI	Prevention of CVS-AKI	Workup and Management of CVS-AKI
**Facilitators**	Jay L. Koyner<br>(Chicago, Il, USA)	Andrew Shaw<br>(Nashville, TN, USA)	Azra Bihorac<br>(Gainesville, FL, USA)	Charles Hobson<br>(Gainesville, FL, USA)
**Members**	Kathleen D. Liu<br>(San Francisco, CA, USA)<br><br>Neesh Pannu<br>(Alberta, Canada)<br><br>Jeffrey B. Rich<br>(Cleveland, OH, USA)<br><br>Lokeswara R. Sajja<br>(Hyderabad, India)	Hrvoje Gasparovic<br>(Zagreb, Croatia)<br><br>Nevin Katz<br>(Baltimore, MD, USA)<br><br>Peter Pickkers<br>(Nijmegen, Netherlands)<br><br>Susana Price<br>(London, UK)<br><br>Zaccaria Ricci<br>(Vicenza, Italy)	Geroge J. Arnaoutakis<br>(Gainesville, FL, USA)<br><br>Daniel T. Engelman<br>(Springfield, MA, USA)<br><br>Charles A. Herzog<br>(Minnesota, MN, USA)<br><br>Fred A. Weaver<br>(Los Angeles, CA, USA)<br><br>Alexander Zarbock<br>(Munster, Germany)	Xiaoqiang Ding<br>(Shanghai, China)<br><br>Kianoush Kashani<br>(Minnesota, MN, USA)<br><br>Ravindra L. Mehta<br>(San Diego, CA, USA)<br><br>Marlies Ostermann<br>(London, UK)

Data S1.

ADQI METHODOLOGY

Our consensus process relied on evidence where available and, in the absence of evidence, consensus expert needed opinion where possible as described previously. Our methods comprise (i) a systematic search for evidence with review and evaluation of the available literature; (ii) the establishment of clinical and physiological outcomes and also measures to be used for comparison of different treatments; (iii) the description of current practice and the rationale for the use of current techniques; and (iv) the analysis of areas in which evidence is lacking and future research is required to obtain new information. The topics chosen for each conference are selected on the basis of the following criteria: (i) prevalence of the clinical problem; (ii) estimate of variation in clinical practice; (iii) potential influence on outcome; (iv) potential for development of evidence-based guidelines; and (v) availability of scientific evidence.

The activities of ADQI conferences are conducted in three stages: (1) pre-conference, (2) conference, and (3) post- conference. Prior to the conference, we identified four topics for discussion pertaining to Cardiac and Vascular associated acute kidney injury (AKI). Conference participants were divided into four working groups, (i. Pathophysiology; ii. Diagnosis and risk assessment; iii. Prevention; and iv. Workup and Management) and tasked with the development of practice recommendations as well as research recommendations where knowledge gaps were recognized. Panelists were assigned to one of the work groups, with one member serving as the group facilitators. Conference directors circulate between the breakout groups and also serve as facilitators and moderators for plenary sessions.

Summary statements were developed through a series of breakout sessions where individual work group members were required to identify key issues for which recommendations were needed and to classify current state of consensus and identify supporting evidence for each issue. Workgroup members were then required to present their findings to the entire group, revising each statement as needed until a final version was agreed upon. The responsibility for presenting the findings of the work group to the rest of the participants was shared by each member on a rotating basis. Group facilitators

revised work group findings as needed after each plenary session. Directives for future research were achieved by asking the participants to: a) identify deficiencies in the literature, b) determine if more evidence is necessary, and c) if so, and articulate general research questions. When possible, pertinent study design issues are also considered. Special observers had a scheduled rotation through each of the four workgroups to provide input. In each breakout session, the workgroups refined the key questions, identified the supporting evidence, and generated recommendations and/or directions for future research as appropriate. Summary statements were developed through these series of alternating breakout and plenary sessions and were further refined until final versions were agreed upon. A writing committee assembled the individual reports from the work groups. Each report was edited to conform to a uniform style and for length. The final reports were mailed to each participant for comment and revision. Once final reports were completed, the writing committee summarized the individual reports into a final conference document that is submitted for publication.

For each group topic studies were identified via MEDLINE, PubMed search, bibliographies of review articles and participants' files. Searches were limited to English language articles. We excluded studies on pediatric population. Each group identified a list of key questions and reviews of the literature were performed in a systematic manner prior to the meeting, as directed by the Workgroup chairs. Although nonrandomized studies were also reviewed, the majority of the Workgroup resources were devoted to review of randomized controlled trials (RCTs), as these were deemed to be most likely to provide data to support level 1 recommendations with very high- or high-quality (A or B) evidence. Exceptions were made by incorporating the best available evidence and expert opinion for topics with sparse evidence. Through consensus of opinion, the available literature was distilled and presented iteratively to the conference participants with the final outputs assessed and aggregated in a plenary session who formally approved the consensus recommendations. The quality of the overall evidence and the strength of recommendations were graded using the Grading of Recommendations Assessment, Development and Evaluation system. Evidence was classified according to levels per EBM methodology.
